# Interactive effects of C-reactive protein levels on the association between *APOE* variants and triglyceride levels in a Taiwanese population

**DOI:** 10.1186/s12944-016-0262-z

**Published:** 2016-05-13

**Authors:** Semon Wu, Lung-An Hsu, Ming-Sheng Teng, Jeng-Feng Lin, Hsin-Hua Chou, Ming-Cheng Lee, Yi-Ming Wu, Cheng-Wen Su, Yu-Lin Ko

**Affiliations:** Department of Life Science, Chinese Culture University, Taipei, Taiwan; Department of Research, Taipei Tzu Chi Hospital, The Buddhist Tzu Chi Medical foundation, New Taipei City, Taiwan; The First Cardiovascular Division, Department of Internal Medicine, Chang Gung Memorial Hospital and Chang Gung University College of Medicine, Taoyuan, Taiwan; The Division of Cardiology, Department of Internal Medicine, Taipei Tzu Chi Hospital, The Buddhist Tzu Chi Medical foundation, 289 Jianguo Road, Xindian District, New Taipei City, 231 Taiwan; School of Medicine, Tzu Chi University, Hualien, Taiwan

**Keywords:** *APOE*, Gene association study, Polymorphism, C-reactive protein (CRP), Gene–trait interaction

## Abstract

**Background:**

Apolipoprotein E (APOE) plays a major role in lipid metabolism and inflammation. However, the association between *APOE* gene polymorphisms and serum triglyceride levels remains controversial. We tested the effects of *APOE* variants on triglyceride levels and their interactions with the inflammatory marker C-reactive protein (CRP) in a Taiwanese population.

**Methods:**

Two APOE single nucleotide polymorphisms (SNPs) rs429358 and rs7412 were genotyped by TaqMan Assay using real time PCR in 595 healthy subjects attending the clinic for routine visits.

**Results:**

After adjustment for clinical covariates, subjects carrying the rs429358-TT genotype and non-ε4 alleles were found to have higher CRP levels, whereas those with rs7412-CC genotype and non-ε2 alleles had significantly higher total and low-density lipoprotein cholesterol levels (all *P* < 0.01). Using subgroup and interaction analyses, we observed significantly lower triglyceride levels in subjects carrying the rs429358-TT genotype and non-ε4 alleles in the low CRP group (*P* = 2.71× 10^−4^ and *P* = 4.32 × 10^−4^, respectively), but not in those in the high CRP group (interaction *P =* 0.013 and 0.045, respectively). In addition, multivariate stepwise linear regression analysis showed that subjects carrying the rs429358-TT genotype and non-ε4 alleles with low CRP levels had significantly lower triglyceride levels (*P* < 0.001 and *P* < 0.001, respectively). In addition, when combined with the risk alleles of *GCKR*, *APOA5* and *LPL* gene variants, we observed that triglyceride levels increased significantly with the number of risk alleles (*P* = 2.9 × 10^−12^).

**Conclusions:**

The combination of SNPs and ε alleles at the *APOE* locus is involved in managing lipid and CRP levels in the Taiwanese population. *APOE* polymorphisms interact with CRP to regulate triglyceride levels, thus triglyceride concentration is influenced by both the genetic background of the *APOE* locus and the inflammatory status of a subject.

**Electronic supplementary material:**

The online version of this article (doi:10.1186/s12944-016-0262-z) contains supplementary material, which is available to authorized users.

## Background

Human apolipoprotein E (APOE) is a 299-amino acid polypeptide responsible for lipid metabolism and cholesterol and triglyceride transport in the blood [1]. APOE is synthesized and secreted by the hepatocytes and is a surface component of primarily triglyceride-rich lipoproteins, such as very low-density lipoproteins (VLDLs), their remnants, chylomicron remnants, and high density lipoproteins (HDLs) [1]. APOE exerts effects by binding to its own receptor and the low-density lipoprotein (LDL) receptor and is the main ligand for clearance of VLDLs and chylomicron remnants, and as such affects circulating concentrations of lipoproteins and plasma levels of cholesterol and triglycerides [2, 3]. Moreover, it was shown that apoE has proinflammatory properties and mediates the presentation of lipid antigens to the immune system and in this way lead to chronic inflammation [4, 5]. In addition, *APOE* transcripts are found in the brain, kidneys, and spleen, suggestive of a crucial function of APOE in these organs [6–8]. *APOE* knockout mice developed severe hypercholesterolemia and accelerated the development of atherosclerotic lesions [9]. In humans, the *APOE* variants are known for their roles in atherosclerosis, cardiovascular disease, Alzheimer disease, aging, and longevity [10–12]. Three APOE isoforms are encoded by three codominant alleles, ε2, ε3, and ε4, which are the direct results of two amino acid substitutions at positions 112 and 158. Therefore, depending on an individual’s genotype, three homozygous (*APOE2/2*, *APOE3/3*, and *APOE4/4*) and three heterozygous (*APOE2/3*, *APOE2/4*, and *APOE3/4*) isoform combinations are present in the human population [13].

Many clinical investigations have shown that serum triglyceride level is an excellent indicator of inflammation, insulin resistance, metabolic syndrome, atherosclerosis, and coronary heart disease (CHD) [14–17]. Previous genome-wide association studies (GWAS) and meta-analyses have mapped the genetic determinants of lipid levels, including that of triglycerides, to several loci enriched with single nucleotide polymorphisms (SNPs) [18]. However, GWAS and fine-mapping studies on the sets of *APOE* SNPs responsible for the lipid traits have yielded conflicting results. Some *APOE* SNPs have been discovered by GWAS; however, fine-mapping studies have suggested a different set of SNPs, which vary substantially in frequency and effect size [18, 19]. Many studies have discovered factors that interact with *APOE* gene polymorphisms in regulating physiological functions. One such factor is C-reactive protein (CRP), an indispensable biomarker for chronic systemic inflammation. Serum CRP levels considerably influenced the relationship between *APOE* SNPs and disease states [20]. Previous studies have found elevated CRP levels associated with hyperlipidemias [21, 22]. CRP gene variants have been independently associated with serum lipid levels [23] and individuals carrying the risk allele for CRP also encounter higher lipid levels and risk of type 2 diabetes [24]. These results suggested CRP may play a role in lipid metabolism.

Since APOE is a multifunctional protein involving lipid metabolism and inflammatory process, this study aimed to investigate the role of *APOE* variants on CRP and lipid levels as well as the interactive effect of CRP levels. Apolipoprotein A-V (*APOA5),* Lipoprotein lipase (*LPL)* and glucokinase regulator (*GCKR)* gene region variants have been consistently associated with serum triglyceride levels [25–27]. The independent effect of *APOE* SNP on triglyceride levels and the genetic risk allele analysis were also investigated with the *LPL*, *GCKR* and *ApoA5* loci variants.

## Methods

### Subjects

In total, 595 patients (312 men, mean age: 44.9 ± 10.4 years; 283 women, mean age: 46.6 ± 10.0 years) were recruited between October 2003 and September 2005at Chang Gung Memorial Hospital. During routine cardiovascular health examinations, the participants answered a questionnaire on medical history and lifestyle characteristics. They underwent physical examinations in which height, weight, waist and hip circumferences, and blood pressure in the sitting position after 15 min of rest were measured. Fasting blood samples, including serum and plasma, were obtained from each patient via the antecubital vein, and were then centrifuged at 3000 × *g* for 15 min at 4 °C. All patients provided informed consent. Exclusion criteria included a history of myocardial infarction, stroke or transient ischemic attack, cancer, current renal or liver disease. Furthermore, to avoid enrolling patients with acute inflammatory disease, those with CRP levels of >10 mg/L [28] and use of lipid-lowering medication relate to variation in CRP and lipid levels were excluded. The clinical and biometrical features of the study population are summarized in Table 1. Current smokers were those who smoked regularly during the survey period. This study was approved by the Ethics Committees of Chang Gung Memorial Hospital (92–315, 6 May 2003).

**Table 1:** Basic characteristics of subjects in this study

	Total (*n* = 595)	Men (*n* = 312)	Women (*n* = 283)	*P* value
Age, yr	45.7 ± 10.2	44.9 ± 10.4	46.6 ± 10.0	0.047
Body mass index, kg/m^2^	24.3 ± 3.4	25.0 ± 3.1	23.5 ± 3.6	<0.001
Glucose (AC), mg/dL	97.2 ± 23.8	99.6 ± 26.4	94.5 ± 20.4	0.01
HDL-C, mg/dL	55.2 ± 14.3	49.7 ± 11.9	61.3 ± 14.2	<0.001
LDL-C, mg/dL	115.9 ± 33.0	118.4 ± 34.0	113.1 ± 31.6	0.051
Total cholesterol, mg/dL	198.5 ± 36.7	200.6 ± 37.3	196.2 ± 36.0	0.14
Triglyceride, mg/dL	142.5 ± 119.2	172.6 ± 147.9	109.3 ± 60.6	<0.001
CRP, mg/L	1.1 ± 1.4	1.1 ± 1.4	1.0 ± 1.4	0.131
Triglyceride/HDL-C ratio	3.01 ± 3.50	3.93 ± 4.42	2.00 ± 1.52	<0.001
SAA, mg/dL	5.4 ± 11.1	6.0 ± 13.3	4.7 ± 8.0	0.161
Fibrinogen, mg/dL	262.4 ± 68.1	260.3 ± 69.9	264.9 ± 66.0	0.405
sICAM1, μg/L	241.2 ± 112.2	243.7 ± 111.4	238.5 ± 113.2	0.579
sVCAM1, μg/L	492.2 ± 133.3	495.7 ± 150.9	488.2 ± 110.9	0.495
MMP2, mg/L	127.3 ± 40.8	124.2 ± 41.4	130.7 ± 40.0	0.056
MMP9, mg/L	142.6 ± 111.8	153.8 ± 115.5	130.2 ± 106.3	0.011
sE-selectin, μg/L	104.2 ± 51.9	117.5 ± 54.9	89.4 ± 44.0	<0.001
Diastolic BP, mmHg	75.0 ± 10.6	77.9 ± 10.4	73.6 ± 10.3	<0.001
Systolic BP, mmHg	114.8 ± 17.6	115.9 ± 16.4	113.7 ± 18.8	0.128
Current smokers, %	25	43.3	4.9	<0.001
Hypertension, %	11.8	15.1	8.1	0.006
Hypercholesterolemia, %	9.6	10.9	8.1	0.157
Diabetes mellitus, %	5	5.8	4.2	0.254

*AC* antecubital, *BP* blood pressure, *CRP* C-reactive protein, *HDL-C* high-density lipoprotein-cholesterol, *LDL-C* low-density lipoprotein-cholesterol, *SAA* serum amyloid A, *sICAM1* soluble intercellular adhesive molecule 1, *sVCAM1* soluble vascular cell adhesive molecule 1, *MMP2* matrix metalloproteinase 2, *MMP9* matrix metalloproteinase 9

Continuous variables are presented as mean ± SD. Triglyceride, HDL-C, LDL-C, total cholesterol, CRP, SAA, sICAM1, sVCAM1, sE-selectin, MMP2 and MMP9 values were logarithmically transformed before statistical testing to meet the assumption of normal distributions; however, the untransformed data are shown. BP levels and lipid variables were analyzed with the exclusion of the subjects using antihypertensive drugs and lipid lowering agents, respectively

### Genomic DNA extraction and genotyping

Genomic DNA was extracted as previously reported [29]. Two *APOE* SNPs, rs429358 (TGC → CGC, Cys112Arg) and rs7412 (CGC → TGC, Arg158Cys), were selected according to the NCBI SNP database (http://www.ncbi.nlm.nih.gov/SNP) for genotyping APOE isoforms and were performed using TaqMan SNP Genotyping Assays from Applied Biosystems (ABI; Foster City, CA, USA). The two *APOE* SNPs were further stratified into three common alleles, ɛ2, ɛ3 and ɛ4: ɛ2, T-T (Cys-Cys); ɛ3, T-C (Cys-Arg); and ɛ4, C-C (Arg-Arg) [13]. Genotyping for *GCKR* rs1260326 and *LPL* rs13702 polymorphisms were also performed using TaqMan SNP Genotyping Assays from Applied Biosystems. Genotyping for *APOA5* rs662799 was performed with polymerase chain reaction and restriction enzymes digestion. Genotyping data are shown in Additional file 1: Table S1. For quality control purposes, approximately 10 % of the samples were re-genotyped in a blinded fashion and the same results were obtained.

### Assays

The levels of most markers, including serum CRP, serum amyloid A (SAA), soluble intercellular adhesive molecule (sICAM1), soluble vascular cell adhesive molecule (sVCAM1), soluble E-selectin (sE-selectin), and matrix metalloproteinase 9 (MMP-9), were measured using a sandwich enzyme-linked immunosorbent assay (ELISA) developed in-house. All in-house kits showed excellent correlation when compared with commercially available ELISA kits [30–32]. Circulating serum matrix metalloproteinase 2 (MMP-2) levels were measured using a commercially available ELISA kit (R&D; Minneapolis, MN, USA). Plasma glucose levels were measured in a central laboratory as previously reported [33]. Total cholesterol and triglyceride levels were measured through automatic enzymatic colorimetry. High-density lipoprotein cholesterol (HDL-C) levels were measured enzymatically after phosphotungsten and magnesium precipitation. Low-density lipoprotein cholesterol (LDL-C) levels were measured using the Friedewald formula for patients with triglyceride levels of <400 mg/dL. When triglyceride levels were >400 mg/dL, serum LDL-C levels were measured with commercial reagents following standard protocol. Plasma fibrinogen levels were measured using the Clauss method adapted for a Sysmex CA1-1500 instrument in the clinical hematology laboratory.

### Statistical analyses

The chi-square test was used to examine differences in the distribution of categorical data including smoking status, hypertension, hypercholesterolemia, and diabetes mellitus. Clinical characteristics that were continuous variables were expressed as means ± SD and tested using the two-sample *t* test or analysis of variance (ANOVA). A generalized linear model was used to analyze lipid and inflammatory marker levels regarding investigated genotypes and confounders. Dominant models were used for numeric association tests after recoding our SNPs from categorical variables to continuous variables, such as 0 and 1 of a particular allele. Triglyceride, HDL-C, LDL-C, total cholesterol, CRP, SAA, sICAM1, sVCAM1, sE-selectin, MMP-2, and MMP-9 level values were logarithmically transformed before statistical analyses to follow a normality assumption. *P* < 0.05, obtained using two-sided tests, was considered statistically significant. Interactions between each SNP, the levels of lipid traits or CRP, and the CRP status were tested using two-way ANOVA. When interaction terms were significant, stratified analyses of the *APOE* genotypes (i.e., genotypes affected by the CRP status) and lipid or CRP levels were performed to further study interactive effects while controlling for other variables including age, smoking status, BMI, and antihypertensive and antidiabetic medication use. In addition, stepwise linear regression analysis was performed to determine independent predictors of triglyceride levels. Deviation from the Hardy–Weinberg equilibrium (HWE), the linkage disequilibrium between polymorphisms, the association of genotypes with lipid or CRP levels, and genotype–CRP status interactions were investigated using Golden Helix SVS Win32 7.3.1 software (Golden Helix). The genetic risk alleles were created to assume each SNP to be independently associated with triglyceride levels (i.e., no interaction between SNPs). The count model assumed each SNP in the panel contributed equally to triglyceride levels, and the genetic risk allele was determined by a simple summation of the number of risk alleles from the *GCKR*, *APOA5*, *LPL* SNPs and *APOE* − CRP subgroups.

## Results

### Characteristics of clinical biochemical factors and biomarkers

Demographic data, clinical biochemical data, and lipid and inflammatory biomarker profiles of the subjects, stratified by sex, are summarized in Table 1. A significantly higher percentage of the men were current smokers (*P* < 0.001) and hypertensive (*P* = 0.006). In addition, the men had significantly higher body mass index (BMI) (*P* < 0.001), diastolic blood pressure (*P* < 0.001), fasting plasma glucose (*P* = 0.01), triglyceride (*P* < 0.001), sE-selectin (*P* < 0.001), and MMP-9 (*P* = 0.011) levels than the women did. By contrast, HDL-C levels (*P* < 0.001) were lower in the men than in the women. No significant deviation from the HWE was observed for the studied polymorphisms (Additional file 1: Table S1, *P* = 0.812 and 0.935 for rs429358 and rs7412, respectively).

### Relationship between APOE SNPs and lipid biomarkers

To determine the effects of *APOE* SNPs on lipid levels, we created an additive model using four lipid traits as variables of interest. After adjustment for age, sex, smoking status, BMI, and antihypertensive and antidiabetic treatments, subjects carrying rs429358-TT genotype had lower LDL-C levels and higher HDL-C levels than those carrying rs429358-CT and –CC genotypes did (Table 2, *P* = 0.025 and 0.040, respectively). The subjects carrying rs429358-CT genotypes had the highest triglyceride levels compared to that of the other two carriers (*P* = 0.034). Among rs7412 carriers, subjects carrying rs7412-CC genotype had the highest total cholesterol and LDL-C levels compared to those carrying the rs7412-CT and TT genotypes (Table 2, *P* = 3.01× 10^−5^ and 4.55× 10^−9^, respectively). Using a recessive model, we discovered that subjects carrying rs429358-CT and –CC genotypes had higher LDL-C and triglyceride levels than those carrying the rs429358-TT genotype did (*P* = 0.043 and 0.027, respectively), whereas subjects carrying rs7412-CT and TT genotypes had lower total cholesterol and LDL-C levels compared to those carrying rs7412-CC genotype (*P* = 3.2 × 10^−5^ and 3.1 × 10^−8^, respectively), which largely confirmed most of the findings from the additive model.

**Table 2:** Levels of lipids and CRP among different *APOE* genotype carriers

*APOE* SNP	MM	Mm	mm	β (95 % CI)	*P* value^a^	MM	Mm + mm	β (95 % CI)	*P* value^a^
rs429358	TT (*n* = 480)	CT (*n* = 96)	CC (*n* = 8)			TT (*n* = 480)	CT + CC (*n* = 104)		
T-cholesterol (mg/dL)	197.1 ± 35.9	204.2 ± 40.1	210.9 ± 28.2	6.67 (−0.13–13.46)	0.054	197.1 ± 35.9	204.7 ± 39.3	7.03 (−0.56–14.63)	0.069
LDL-C (mg/dL)	114.5 ± 32.4	121.0 ± 35.7	132.1 ± 23.0	7.02 (0.90–13.14)	0.025	114.5 ± 32.4	121.8 ± 34.9	7.06 (0.22–13.91)	0.043
HDL-C (mg/dL)	55.8 ± 14.7	52.3 ± 12.1	51.3 ± 13.3	−0.29 (−0.038 to −0.001)	0.040	55.8 ± 14.7	52.2 ± 12.1	−0.02 (−0.04–0.001)	0.063
Triglyceride (mg/dL)	138.3 ± 119.9	165.1 ± 122.7	138.0 ± 68.5	0.047 (0.004–0.09)	0.034	138.3 ± 119.9	163.0 ± 119.4	0.055 (0.006–0.103)	0.027
CRP (mg/L)	1.12 ± 1.40	0.79 ± 1.19	1.96 ± 1.99	−0.12 (−0.21 to −0.04)	0.005	1.12 ± 1.40	0.88 ± 1.29	−0.17 (−0.27 to −0.08)	3.8 x 10^−4^
rs7412	CC (*n* = 496)	CT (*n* = 84)	TT (*n* = 5)			CC (*n* = 496)	CT + TT (*n* = 89)		
T-cholesterol (mg/dL)	201.0 ± 35.5	184.3 ± 37.9	169.8 ± 65.8	−15.77 (−23.13 to −8.40)	3.01 x 10^−5^	201.0 ± 35.5	183.5 ± 39.5	−17.08 (−25.08–9.08)	3.2 x 10^−5^
LDL-C (mg/dL)	118.9 ± 32.1	100.2 ± 31.8	65.0 ± 21.4	−19.82 (−26.36 to −13.28)	4.55 x 10^−9^	118.9 ± 32.1	98.3 ± 32.3	−20.36 (−27.49–13.24)	3.1 x 10^−8^
HDL-C (mg/dL)	54.7 ± 14.2	57.8 ± 15.3	50.4 ± 8.5	0.01 (−0.007–0.34)	0.188	54.7 ± 14.2	57.4 ± 15.0	0.02 (−0.005–0.04)	0.129
Triglyceride (mg/dL)	143.6 ± 121.1	133.2 ± 108.9	217.8 ± 176.1	0.001 (−0.046–0.049)	0.957	143.6 ± 121.1	137.9 ± 113.9	−0.009 (−0.06–0.04)	0.730
CRP (mg/L)	1.08 ± 1.33	1.10 ± 1.67	0.75 ± 0.68	−0.12 (−0.11–0.08)	0.709	1.08 ± 1.33	1.08 ± 1.63	−0.01 (−0.11–0.09)	0.795

*CI* confidence interval, *M* major allele, *m* minor allele

^a^The multiple linear regressions were adjusted for age, sex, BMI, current smoking status, and anti-hypertensive and anti-diabetic treatments

### Association between APOE polymorphisms and inflammatory biomarkers

To determine whether the *APOE* genotypes influenced any inflammatory marker, we analyzed the levels of eight inflammatory markers, CRP, fibrinogen, SAA, sICAM1, sVCAM1, sE-selectin, MMP-2, and MMP-9. Except for CRP levels, no significant differences were observed in inflammatory marker levels among the *APOE* genotypes (Additional file 1: Table S2). Table 2 shows variation in serum CRP levels across the *APOE* genotypes. After an additive model was adjusted for age, sex, smoking status, BMI, and antihypertensive and antidiabetic medication use, only rs429358 genotypes was significantly associated with CRP levels (*P* = 0.005). In addition, a dominant model adjusted for age, sex, smoking status, BMI, and antihypertensive and antidiabetic medication use revealed that subjects carrying rs429358-CC and -CT genotypes were associated with lower CRP levels than those carrying TT genotype did (*P* = 3.8 × 10^−4^) (Table 2). In contrast, none of the rs7412 genotypes were associated with CRP levels.

### APOE allele combinations, lipids, and CRP

We assigned *APOE* allele combinations into three groups, ε2 (ε2ε2 and ε2ε3), ε3 (ε3ε3), and ε4 (ε4ε4 and ε4ε3), and used multiple linear regressions to analyze their effects on lipid and CRP levels. After adjustment for age, sex, smoking status, BMI, and antihypertensive and antidiabetic treatments using an additive model, different allele groups were shown as exerting significant differences in the levels of total cholesterol, LDL-C, HDL-C, and CRP (Table 3, *P* = 4.24× 10^−6^, 1.94× 10^−7^, 0.008, and 0.018, respectively). The ε2 carriers had significantly lower total cholesterol and LDL-C levels than the non-ε2 carriers did (*P* = 6.65 × 10^−6^ and 5.12 × 10^−9^, respectively). The ε4 carriers had significantly higher LDL-C and triglyceride levels (*P* = 0.031 and 0.018, respectively) and lower HDL-C and CRP levels than the non-ε4 carriers did (*P* = 0.020 and 2.12× 10^−4^, respectively).

**Table 3:** Levels of lipids and CRP among different *APOE* ε genotypes carriers

*APOE* ε genotypes	T-cholesterol (mg/dL)	Beta (95 % CI)	*P* value^a^	LDL-C (mg/dL)	Beta (95 % CI)	*P* value^a^	Triglyceride (mg/dL)	Beta (95 % CI)	*P* value^a^	HDL-C (mg/dL)	Beta (95 % CI)	*P* value^a^	CRP (mg/L)	Beta (95 % CI)	*P* value^a^
ε2 (*n* = 81)	181.7 ± 36.2	0.03 (0.01–0.04)	4.24 × 10^−5^	96.5 ± 27.6	0.05 (0.03–0.07)	1.94 × 10^−7^	138.4 ± 114.1	0.03 (−0.003–0.06)	0.054	57.3 ± 15.2	−0.02 (−0.03 to −0.002)	0.008	1.09 ± 1.70	−0.08 (−0.14 to −0.01)	0.018
ε3 (*n* = 399)	200.2 ± 35.1			118.1 ± 32.1			138.3 ± 121.1			55.5 ± 14.7			1.13 ± 1.33		
ε4 (*n* = 96)	205.0 ± 36.9			122.3 ± 32.0			165.5 ± 119.7			51.7 ± 11.8			0.88 ± 1.33		
ε2 (*n* = 81)	181.7 ± 36.2	0.05 (0.03–0.06)	6.65 × 10^−6^	96.5 ± 27.6	0.09 (0.06–0.12)	5.12 × 10^−9^	138.4 ± 114.1	0.01 (−0.05–0.06)	0.779	57.3 ± 15.2	−0.02 (−0.04–0.01)	0.172	1.09 ± 1.70	0.02 (−0.09–0.12)	0.782
Non-ε2 (*n* = 495)	201.1 ± 35.5			118.9 ± 32.1			143.6 ± 121.2			54.8 ± 14.2			1.08 ± 1.33		
ε4 (*n* = 96)	205.0 ± 36.9	0.02 (−0.002–0.03)	0.068	122.3 ± 32.0	0.03 (0.004–0.06)	0.031	165.5 ± 119.7	0.06 (0.01–0.11)	0.018	51.7 ± 11.8	−0.02 (−0.04 to −0.001)	0.020	0.88 ± 1.33	−0.19 (−0.28 to −0.09)	2.12 × 10^−4^
Non-ε4 (*n* = 480)	197.1 ± 35.9			114.5 ± 32.4			138.3 ± 119.9			55.8 ± 14.7			1.12 ± 1.40		

ε2 (ε2ε2 and ε2ε3); ε3 (ε3ε3); ε4 (ε3ε4 and ε4ε4)

^a^The multiple linear regressions were adjusted for age, sex, BMI, current smoking status, and anti-hypertensive and anti-diabetic treatments

### APOE and CRP interaction

**Fig. 1:**
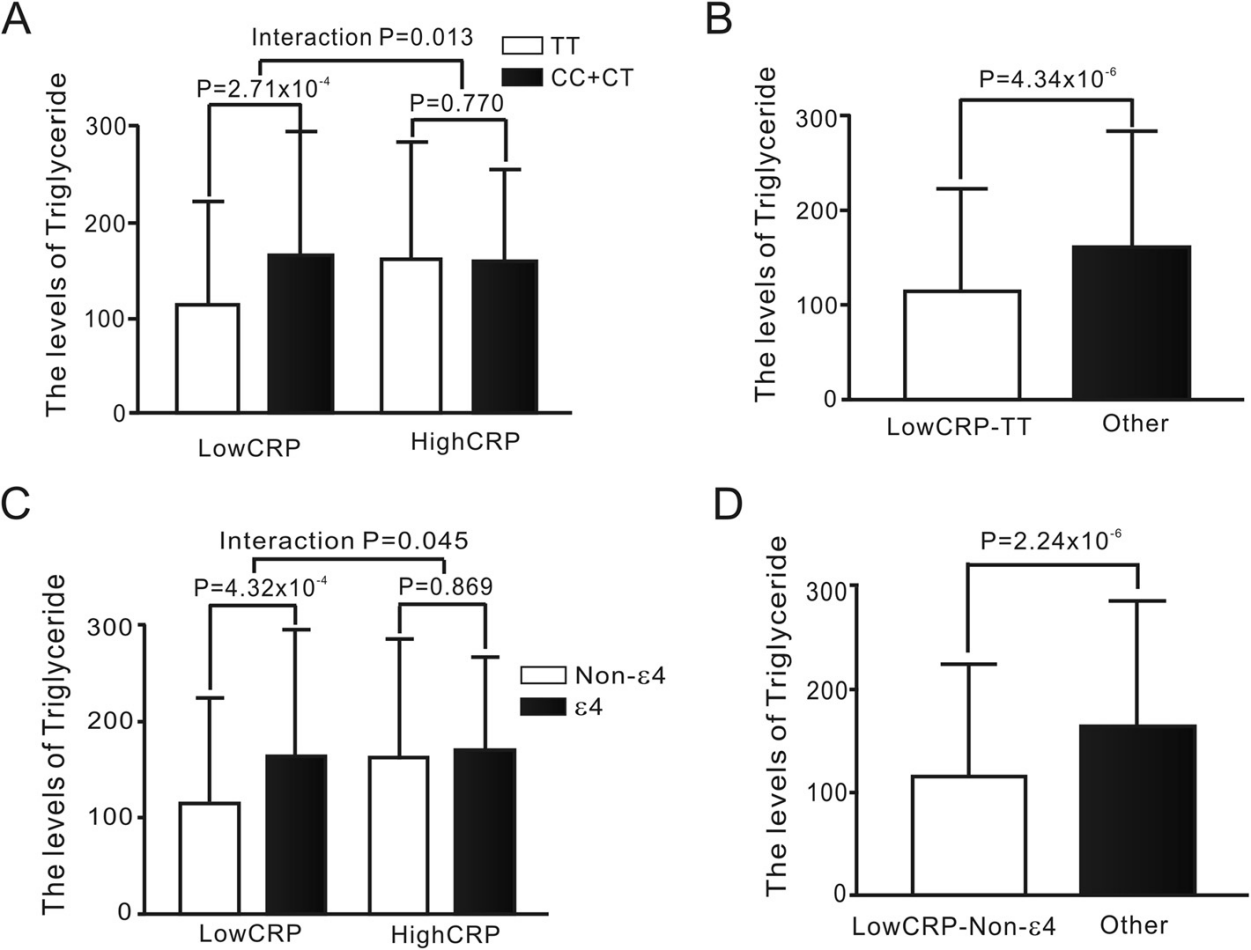
The triglyceride levels according to rs429358 genotypes **a**, **b** and *APOE* isoforms **c**, **d** in Taiwanese subjects in high and low C-reaction protein (CRP) subgroups. **a** After adjusting for clinical covariates, subjects carrying the *APOE* rs429358-TT genotype had lower triglyceride levels compared to those carrying non-TT genotypes in the low CRP subgroup but not in the high CRP subgroup. **b** Significantly lower triglyceride levels were also noted when subjects with the *APOE* rs429358-TT genotype and low CRP levels were compared to the other subjects of the study. **c**, **d** Similar results were also noted when triglyceride levels from carriers of the *APOE* ε4 and non-ε4 isoforms were compared. Low CRP level was defined as CRP levels lower or equal to 0.62 mg/L, whereas high CRP level was defined as CRP levels higher than 0.62 mg/L

Because we were interested in the effects of CRP–*APOE* interaction on lipid levels, we divided the subjects according to their CRP levels and *APOE* genotypes, and analyzed the triglyceride levels among different *APOE* genotypes and allele carriers in the low and high CRP groups (Fig. 1). After adjustment for clinical covariates, ε4 carriers exhibited significantly higher triglyceride levels exclusively in the low CRP group (*P* = 4.32× 10^−4^), and interaction was observed between CRP levels and different *APOE* allele carrier groups (interaction *P* = 0.045). Similarly, subjects carrying the rs429358-CC and CT genotypes had higher triglyceride levels than those carrying rs429358-TT genotypes did in the low CRP group (*P* = 2.71× 10^−4^). None of these observations were seen in the high CRP group.

### Stepwise regression on triglyceride levels

**Fig. 2:**
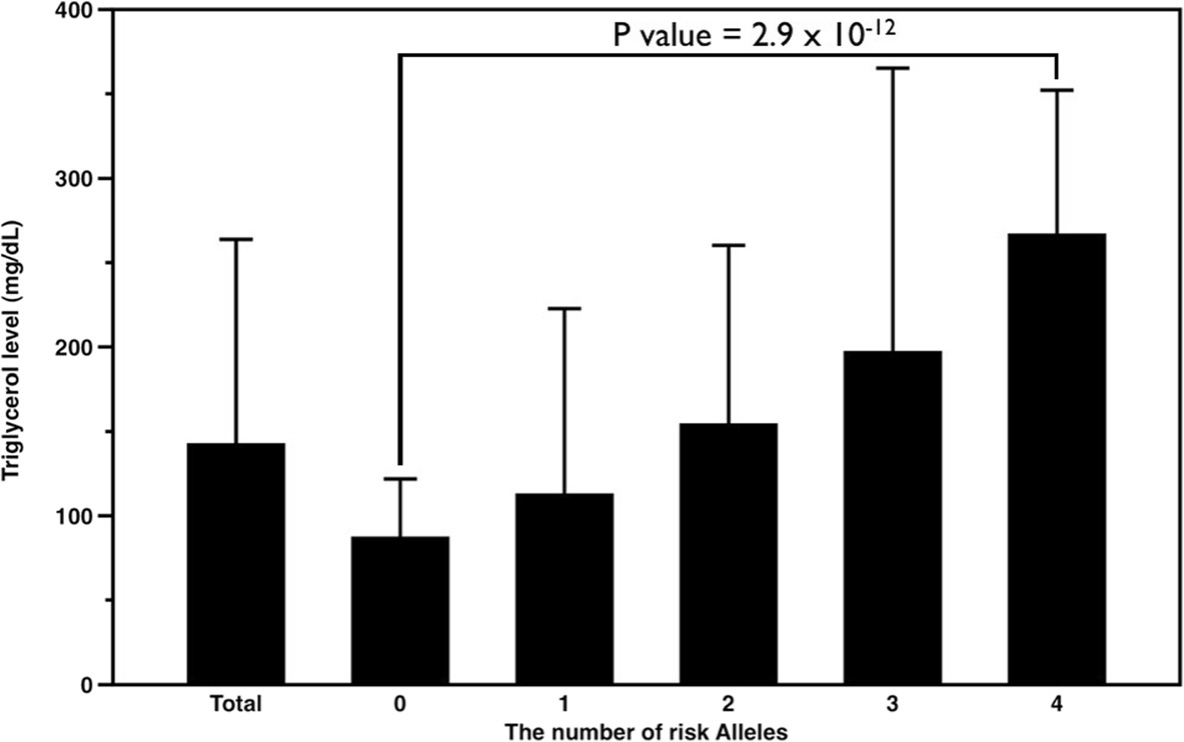
The triglyceride levels according to number of risk alleles calculated from number of SNPs of the *GCKR*, *APOA5*, and *LPL* genes, and the APOE − CRP subgroups. The triglyceride levels were as follows: no risk allele (2.3 % of the population): 87.7 ± 34.2 mg/dL; one risk allele (40.8 % of the population): 113.3 ± 109.3 mg/dL; two risk alleles (42.0 % of the population): 154.9 ± 105.3 mg/dL; three risk alleles (14.4 % of the population): 197.6 ± 167.5 mg/dL; three or four risk alleles (0.5 % of the population): 267.3 ± 84.7 mg/dL

Because we observed an interaction between *APOE* genotypes and CRP, we next studied the effects of *APOE* − CRP subgroups on triglyceride by using stepwise regression to adjust for smoking status, BMI, age, and the three *LPL*, *GCKR* and *ApoA5* SNPs that were independent variables associated with triglyceride levels in previous studies [25–27]. Our results also showed that the studies *LPL*, *GCKR* and *ApoA5* SNPs were significantly associated triglyceride levels (Additional file 1: Table S3). The effect of the non-ε4 − low CRP subgroup on triglyceride levels was significant (β: 0.087, *P* < 0.001) (Table 4). Similar results were observed for the rs429358-TT − low CRP subgroup (β: 0.081, *P* < 0.001) (Table 4). When CRP levels were included in the independent variables, the effect of the non-ε4 − low CRP or rs429358-TT − low CRP subgroup on triglyceride levels was still significant (data not shown). We next assayed the combined effects of risk alleles of *GCKR*, *APOA5*, *LPL*, and *APOE* − CRP subgroups on triglyceride levels (Fig. 2). We observed that triglyceride levels increased significantly with the number of risk alleles (*P* = 2.9 × 10^−12^).

**Table 4:** Triglyceride levels: stepwise linear regression analysis, including genotypes

Variable	Beta	R^2a^	*P* value	Beta	R^2a^	*P* value
Current smokers	0.140	0.105	<0.001	0.130	0.107	<0.001
BMI	0.016	0.180	<0.001	0.016	0.183	<0.001
*APOE* non-ε4–Low CRP	-	-	-	0.087	0.213	<0.001
*APOE* rs429358-TT–Low CRP	0.081	0.209	<0.001	-	-	-
sex	−0.087	0.234	<0.001	−0.086	0.239	<0.001
GCKR rs1260326-TT	−0.071	0.249	0.001	−0.072	0.253	0.001
APOA5 rs662799-GG	−0.114	0.262	0.001	−0.104	0.265	0.003
LPL rs13702-GG	0.118	0.272	0.004	0.119	0.276	0.004
age	0.002	0.278	0.029	-	-	-

^*a*^Cumulative R^2^

The multiple linear regressions were adjusted for age, gender, smoking status, BMI, anti-hypertensive and anti-diabetic treatments, the *APOE* rs429358 genotypes, the *APOA5* rs662799 genotypes, the *GCKR* rs1260326 genotypes and the *LPL* rs13702 genotypes. Low CRP subgroups: subjects with CRP levels lower or equal to 0.62 mg/L

## Discussion

This investigation analyzed the association and interaction among *APOE* genotypes, various lipids including triglycerides, and the inflammatory marker CRP in a Taiwanese population. First, regarding the relationship between *APOE* SNPs and lipid levels, our data revealed significant associations between various genotypes or allelic combinations of the *APOE* SNP rs7412 and total cholesterol and LDL-C levels. Borderline significance (i.e., 0.01 < *P* < 0.05) was noted between various rs429358 genotypes and triglyceride, LDL-C, and HDL-C levels. Regarding the relationship between various *APOE* ε alleles and lipid levels, we observed significant differences in total cholesterol, LDL-C, and HDL-C levels among the carriers of the three *APOE* ε allele groups. In addition, when ε2 and ε4 were analyzed, we found significant differences in total cholesterol and LDL-C levels between the ε2 and non-ε2 carriers. Second, regarding the relationship between *APOE* and CRP, our data revealed significant associations between various alleles or allelic combinations of the *APOE* SNP rs429358 and CRP levels and a significant difference in CRP levels between the ε4 and non-ε4 carriers. Finally, an interaction was discovered between CRP and *APOE*, and differences in triglyceride levels were clearly evident between the carriers of different *APOE* genotypes when stratified by low CRP status. In univariate and multivariate analyses, significant differences were found in triglyceride levels in carriers of the rs429358-TT genotype or non-ε4 alleles from the low CRP group compared with the triglyceride levels in the other carriers. The association between *APOE*-CRP subgroups and triglyceride levels was independent of SNPs of *APOA5*, *GCKR*, and *LPL*. However, these risk alleles of *APOA5*, *GCKR*, and *LPL* interacted with *APOE*-CRP subgroups synergistically to increase triglyceride levels. These results will certainly increase our understanding of the involvement of APOE in cardiovascular diseases.

APOE plays a major role in lipid metabolism and inflammatory response. Our results regarding lipid metabolism echo those of past studies that have shown that ε4 carriers have high total cholesterol and LDL-C levels [34–36]. Regarding inflammation, our results also support previous results showing that ε4 carriers have low CRP levels [37–40]. In addition to these genotype-dependent effects on lipids and CRP levels, ε4 is associated with infectious diseases [41] and a high risk of age-related cardiovascular and Alzheimer disease [42, 43]. The differential regulation of lipids and CRP, in particular by *APOE* ε4, could be explained by a structural change caused by a cysteine-to-arginine substitution at residue 112 [37], down regulation of the mevalonate/cholesterol synthetic pathway in ε4 carriers [39], or different patterns of linkage disequilibrium between ε4 and other SNPs that are yet to be characterized [44].

The association between *APOE* polymorphisms and triglyceride levels is particularly complicated because many studies, such as our initial comparison among the ε2, ε3, ε4 carriers, yield negative results. Nevertheless, some studies have reported positive findings, particularly for the ε4 variant. Dong et al. (2013) showed that ε4 was associated with higher triglyceride levels in patients with renal disease [35]. Carvarlho-Wells et al. (2010) reported that ε4 carriers have higher postprandial triglyceride levels only in adults aged >50 years [45]. The results of both studies are consistent with our finding that ε4 carriers have slightly higher triglyceride levels than non-ε4 carriers do. Maxwell et al. (2013) reported that though ε4 negatively affects the hazard ratio of CHD divided by triglycerides, *APOE* genotypes, overall, do interact with triglycerides to increase the risk of CHD in a linear model of a pooled European-American population, but not an African-American population [46]. According to these studies, each APOE isoform seems to interact with triglyceride metabolism differently, and under various physiological conditions (i.e., age, ethnicity, disease states, and subcategories), these interactions affect triglyceride levels and contribute to the differential odds of the same disease in a multifactorial manner. This may explain why observed odds for coronary disease among ε4 carriers decreases when subjects with different backgrounds were pooled into a substantial sample for meta-analysis [47]. Although we did not measure odd ratios of any disease in this study, an increased difference was observed in triglyceride levels between the ε4 and non-ε4 carriers in the low CRP group, thus providing an acceptable example of the inconsistent regulation of the triglyceride trait. Furthermore, previous studies suggested that the genetic profiles such as the genotype score may be useful for the early detection and treatment of dyslipidemias and triglyceride levels [48, 49]. We also found a cumulative effect in common risk alleles for triglyceride levels. There was a significant association between triglyceride levels and an increasing genotype number of risk alleles. These findings indicated that multiple variants might act together to influence the development of various phenotypes and disease.

Studies have investigated the gene − environment interaction of gene variants on various traits. Qi et al. (2009) reported that the variants of the interleukin-6 receptor SNP rs8192284 modified diabetes risk in women with different CRP levels, and the highest risk was observed in those with the subjects carrying rs8192284-AA genotype in the highest CRP quartile [50]. Our findings provide another example of complex gene–environment interaction, with the subjects carrying *APOE* rs429358-TT genotype and non-ε4 alleles substantially reducing the levels of triglyceride in subjects with lower CRP concentration. The regulation of triglyceride levels is associated with inflammation and insulin resistance; therefore, increased triglyceride levels for both the ε4 and non-ε4 carriers in the high CRP group are acceptable. Unexpectedly, triglyceride levels remained elevated for the ε4 carriers with a low CRP level. Although an association was observed between ε4 and decreased CRP levels [40, 51], ε4 exhibited proinflammatory activities [52]. Our finding thus suggests that ε4 may, possibly through an alternative pro-inflammatory pathway, regulate triglyceride independently of CRP. By contrast, CRP levels might be upregulated by some proinflammatory factors, directly or indirectly masking the influence of ε4. For example, hs-CRP levels were not considerably different between *APOE* genotype carriers in Moroccan patients with end-stage renal disease (ESRD) [53], and, in our study, some ε4 carriers exhibited high CRP levels. Although the cause of high CRP levels regardless of *APOE* genotypes in our sample population is unknown, we suspect that certain health conditions (e.g., ESRD) are responsible for this phenomenon. Further investigation is required to address this possible association.

We propose that the CRP − APOE − triglyceride triad might be a common motif of information processing that bridges clinical manifestations of genetic variants to outputs of signaling units in the body (e.g., systemic inflammation) and generates pleiotropic phenotypes. Its relationship with CHD states requires further investigation, and discovering more loci, covariates, or traits that interact with APOE in a relationship quantitative trait loci pattern [46, 54, 55] might be insightful.

## Conclusion

In conclusion, our data revealed a CRP-dependent association between *APOE* polymorphisms and triglyceride levels. *APOE* ε4 alleles along with low CRP levels were an independent determinant of triglyceride levels in our multivariate regression analysis. CRP is considered an inflammatory marker, thus we propose that the effects of *APOE* genotypes on triglyceride levels in the blood might be associated with the inflammatory status of an individual.

### Limitations

One limitation of this study is the relatively low number of subjects genotyped; replication of the current results in a second cohort would support the strength of the study. Independent association studies with larger sample size and functional data are required to confirm our results before any definitive conclusions can be drawn. Another limitation is the study’s cross-sectional design, which means that the results may be used to draw only limited inference regarding the relationship between exposure and outcome.

## Additional file

Additional file 1: Table S1. Primer sequences and restriction enzymes (RE) used in all gene polymorphisms. **Table S2**. Analysis of inflammation markers with *APOE* genotypes. **Table S3**. Associations between different SNPs and triglyceride levels (mg/dL). (DOCX 21 kb)

## Abbreviations

AC: antecubital
APOA5: apoliporotein A-V
APOE: Apolipoprotein E
BMI: body mass index
BP: blood pressure
CHD: coronary heart disease
CRP: C-reactive protein
ESRD: end-stage renal disease
GCKR: glucokinase regulator
GWAS: genome-wide association studies
HDL-C: high-density lipoprotein-cholesterol
LDL-C: low-density lipoprotein-cholesterol
LPL: lipoprotein lipase
MMP2: matrix metalloproteinase 2
MMP9: matrix metalloproteinase 9
SAA: serum amyloid A
sICAM1: soluble intercellular adhesive molecule 1
SNP: single nucleotide polymorphism
sVCAM1: soluble vascular cell adhesive molecule 1
