# Supplementary material for: Interactive effects of C-reactive protein levels on the association between APOE variants and triglyceride levels in a Taiwanese population
Source: Lipids Health Dis. 2016 May 13;15:94. doi: 10.1186/s12944-016-0262-z (PMC4866423; doi:10.1186/s12944-016-0262-z)
Supplement: Additional file 1: Table S1. — Primer sequences and restriction enzymes (RE) used in all gene polymorphisms. Table S2. Analysis of inflammation markers with APOE genotypes. Table S3. Associations between different SNPs and triglyceride levels (mg/dL). (DOCX 21 kb) [file 12944_2016_262_MOESM1_ESM.docx]

Supplementary Table 1. Primer sequences and restriction enzymes (RE) used in all gene polymorphisms.

| Gene and SNP number | genotyping | Allele | Location | H-W P value |
| --- | --- | --- | --- | --- |
| *APOE* rs429358 | TaqMan SNP Genotyping Assays | C/T | exon 4 missense (Cys→Arg) | 0.812 |
| *APOE* rs7412 | TaqMan SNP Genotyping Assays | C/T | exon 4 missense (Arg→Cys) | 0.935 |
| *APOA5 rs662799* | 5'-GATTGATTCAAGATGCATTTAGGAC-3' (F)  5'-CCCCAGGAACTGGAGCGAAATT-3' (R)  *MseI* | A/G | upstream variant | 0.519 |
| *GCKR rs1260326* | TaqMan SNP Genotyping Assays | C/T | exon 15 | 0.899 |
| *LPL rs13702* | TaqMan SNP Genotyping Assays | A/G | 3’UTR | 0.601 |

Derived or predicted from data obtained from the NCBI SNP databank.

Supplementary Table 2 .Analysis of inflammation markers with *APOE* genotypes

| Inflammation markers | *APOE* rs429358 | | | *APOE* rs7412 | | |
| --- | --- | --- | --- | --- | --- | --- |
|  | genotypes | Mean ± SD | P value^#^ | genotypes | Mean ± SD | P value^#^ |
| SAA (mg/L) | CC (n = 8) | 20.45 ± 42.35 | 0.070 | CC (n = 487) | 5.06 ± 9.69 | 0.889 |
|  | CT (n = 95) | 5.92 ± 14.56 |  | CT (n = 83) | 7.19 ± 17.61 |  |
|  | TT (n = 471) | 4.99 ± 8.87 |  | TT (n = 5) | 3.60 ± 3.28 |  |
|  | Total (n = 574) | 5.36 ± 11.16 |  | Total (n = 575) | 5.35 ± 11.15 |  |
| Fibrinogen (mg/dL) | CC (n = 8) | 285.6 ± 97.40 | 0.542 | CC (n = 496) | 263.7 ± 69.49 |  |
|  | CT (n = 96) | 256.3 ± 71.57 |  | CT (n = 84) | 255.4 ± 54.85 | 0.737 |
|  | TT (n = 480) | 263.5 ± 66.73 |  | TT (n = 5) | 277.3 ± 110.3 |  |
|  | Total (n = 584) | 262.6 ± 67.98 |  | Total (n = 585) | 262.7 ± 67.93 |  |
| VCAM1 (μg/L) | CC (n = 8) | 566.5 ± 161.7 | 0.693 | CC (n = 492) | 495.8 ± 139.1 | 0.464 |
|  | CT (n = 95) | 490.9 ± 106.2 |  | CT (n = 83) | 477.6 ± 102.7 |  |
|  | TT (n = 476) | 492.0 ± 138.5 |  | TT (n = 5) | 454.8 ± 61.72 |  |
|  | Total (n = 579) | 492.9 ± 134.2 |  | Total (n = 580) | 492.8 ± 134.1 |  |
| MMP2 (mg/L) | CC (n = 8) | 133.2 ± 28.36 | 0.793 | CC (n = 492) | 127.0 ± 42.57 | 0.881 |
|  | CT (n = 95) | 124.8 ± 46.98 |  | CT (n = 83) | 128.6 ± 31.31 |  |
|  | TT (n = 476) | 127.5 ± 39.92 |  | TT (n = 5) | 123.0 ± 12.15 |  |
|  | Total (n = 579) | 127.1 ± 40.99 |  | Total (n = 580) | 127.2 ± 40.96 |  |
| MMP9 (mg/L) | CC (n = 8) | 119.2 ± 38.83 | 0.471 | CC (n = 485) | 143.8 ± 114.4 | 0.809 |
|  | CT (n = 94) | 155.4 ± 140.9 |  | CT (n = 82) | 135.6 ± 103.4 |  |
|  | TT (n = 469) | 140.2 ± 106.6 |  | TT (n = 5) | 152.5 ± 66.94 |  |
|  | Total (n = 571) | 142.4 ± 112.4 |  | Total (n = 572) | 142.7 ± 112.4 |  |
| ICAM1 (μg/L) | CC (n = 8) | 255.0 ± 121.3 | 0.757 | CC (n = 491) | 242.7 ± 117.9 | 0.839 |
|  | CT (n = 95) | 246.2 ± 123.7 |  | CT (n = 83) | 233.3 ± 75.61 |  |
|  | TT (n = 475) | 240.6 ± 110.5 |  | TT (n = 5) | 267.8 ± 109.7 |  |
|  | Total (n = 578) | 241.7 ± 112.8 |  | Total (n = 579) | 241.6 ± 112.7 |  |
| sE-selectin (μg/L) | CC (n = 8) | 119.3 ± 67.18 | 0.907 | CC (n = 491) | 104.9 ± 53.17 | 0.830 |
|  | CT (n = 95) | 105.4 ± 54.22 |  | CT (n = 82) | 99.48 ± 43.62 |  |
|  | TT (n = 474) | 103.6 ± 51.05 |  | TT (n = 5) | 109.6 ± 26.43 |  |
|  | Total (n = 577) | 104.1 ± 51.75 |  | Total (n = 578) | 104.1 ± 51.74 |  |

All selection: CRP≦10 & lipid-lowering drugs.

^#^Multiple linear regression, adjusted for age, sex, BMI, current smoking status, and anti-hypertensive and anti-diabetic medication.

Supplementary Table 3. Associations between different SNPs and triglyceride levels (mg/dL).

| **Gene**  **name** | **SNP number** | **genotypes** | **triglyceride levels**  **Means± SD (N)** | ***P* value** |
| --- | --- | --- | --- | --- |
| GCKR | rs1260326 | CC | 134.9 ± 96.4 (161) | 0.004 |
|  |  | CT | 135.7 ± 122.7 (280) |  |
|  |  | TT | 166.3 ± 136.0 (142) |  |
|  |  | CC+CT | 135.4 ± 113.7(441) | 0.001 |
|  |  | TT | 166.3 ± 136.0 (142) |  |
|  |  | CC | 134.9 ± 96.4 (161) | 0.170 |
|  |  | CT+TT | 146.0 ± 128.0(422) |  |
| APOA5 | rs662799 | AA | 125.5 ± 104.8 (287) | 3.28x10^-6^ |
|  |  | AG | 153.7±129.0 (260) |  |
|  |  | GG | 186.8 ± 131.6 (46) |  |
|  |  | AA+AG | 138.9 ± 117.6 (547) | 3.03x10^-4^ |
|  |  | GG | 186.8 ± 131.6 (46) |  |
|  |  | AA | 125.5 ± 104.8 (287) | 9.17x10^-5^ |
|  |  | AG+GG | 158.6 ± 129.7 (306) |  |
| LPL | rs13702 | TT | 149.4 ± 122.5 (366) | 0.001 |
|  |  | TC | 137.4±122.8 (187) |  |
|  |  | CC | 95.4 ± 34.2 (31) |  |
|  |  | TT+TC | 145.3 ± 122.6 (553) | 0.006 |
|  |  | CC | 95.4 ± 34.2 (31) |  |
|  |  | TT | 149.4 ± 122.5 (366) | 0.005 |
|  |  | TC+CC | 131.4 ± 115.4 (218) |  |

N: number of subjects; *P* value adjusted for age, sex, BMI, current smoking status, and anti-hypertensive and anti-diabetic treatments.
